# Detection of the LINE-1 retrotransposon RNA-binding protein ORF1p in different anatomical regions of the human brain

**DOI:** 10.1186/s13100-017-0101-4

**Published:** 2017-11-22

**Authors:** Debpali Sur, Raj Kishor Kustwar, Savita Budania, Anita Mahadevan, Dustin C. Hancks, Vijay Yadav, S. K. Shankar, Prabhat K. Mandal

**Affiliations:** 1 Department of Biotechnology, IIT Roorkee, Roorkee, Uttarakhand India; 2 School of Environmental Sciences, Jawaharlal Nehru University, New Delhi, India; 3 Human Brain Tissue Repository (HBTR), Neurobiology Research Centre, NIMHANS, Bangalore, 560 029 India; 4 Department of Human Genetics, University of Utah, Salt Lake City, UT USA; 5 Present address: Department of Immunology, UT South-western Medical Centre, Dallas, TX USA

**Keywords:** Retrotransposon, LINE-1, ORF1p antibody, Active retrotransposition in human brain, Somatic mosaicism

## Abstract

**Background:**

Recent reports indicate that retrotransposons – a type of mobile DNA – can contribute to neuronal genetic diversity in mammals. Retrotransposons are genetic elements that mobilize via an RNA intermediate by a “copy-and-paste” mechanism termed retrotransposition. Long Interspersed Element-1 (LINE-1 or L1) is the only active autonomous retrotransposon in humans and its activity is responsible for ~ 30% of genomic mass. Historically, L1 retrotransposition was thought to be restricted to the germline; however, new data indicate L1 s are active in somatic tissue with certain regions of the brain being highly permissive. The functional implications of L1 insertional activity in the brain and how host cells regulate it are incomplete. While deep sequencing and qPCR analysis have shown that L1 copy number is much higher in certain parts of the human brain, direct in vivo studies regarding detection of L1-encoded proteins is lacking due to ineffective reagents.

**Results:**

Using a polyclonal antibody we generated against the RNA-binding (RRM) domain of L1 ORF1p, we observe widespread ORF1p expression in post-mortem human brain samples including the hippocampus which has known elevated rates of retrotransposition. In addition, we find that two brains from different individuals of different ages display very different expression of ORF1p, especially in the frontal cortex.

**Conclusions:**

We hypothesize that discordance of ORF1p expression in parts of the brain reported to display elevated levels of retrotransposition may suggest the existence of factors mediating post-translational regulation of L1 activity in the human brain. Furthermore, this antibody reagent will be useful as a complementary means to confirm findings related to retrotransposon biology and activity in the brain and other tissues in vivo.

**Electronic supplementary material:**

The online version of this article (10.1186/s13100-017-0101-4) contains supplementary material, which is available to authorized users.

## Background

Historically, the genome was thought to be identical in every cell throughout an organism except immune cells and germ cells. Notably, the discovery of transposable elements and their mobilization in somatic and germ cells indicates that genomes within an organism are by no means static [1, 2]. Since initial characterization by Barbara McClintock, transposons have been considered as insertional mutagens; in other words transposon activity may result in single-gene disease [1, 3, 4]. Typically considered “selfish” parasitic sequences, recent findings dispute this traditional model and have demonstrated the multifaceted potential of transposons. Indeed, transposons are now appreciated as major players in genome evolution in most organisms including mammals [5].

Along with being widespread across mammalian genomes, Long Interspersed Element −1 (LINE-1 or L1) is the only active autonomous retrotransposon in the modern human genome [6]. In addition, L1 is the most abundant retrotransposon by sequence mass accounting for 17% of the human genome (~500,000 copies) [7, 8]. L1 mobilizes from one genomic location to another using RNA as an intermediate via a process referred to as retrotransposition. Thus, these types of elements are referred to as retrotransposons [6]. Although most of the L1s are inactive due to point mutations, 5′-truncations and other rearrangements including inversions, around 80–100 L1s are active in any given human [9].

An active, full-length L1 is ~6 kb in length. It encodes an internal promoter within a ~900 base pair (bp) 5′-UTR, two open-reading frames termed ORF1 and ORF2 separated by a small inter-ORF spacer and a 3'-UTR (~205 bp). Genomic insertions end in a polyA sequence derived from the mRNA polyA tail (~40-120 bp) and are flanked by direct repeats of varying length known as target-site duplications (TSD, typically 4–20 bp in length) at the site of insertion [4, 6, 10]. ORF1 encodes a protein (ORF1p) with single-stranded nucleic acid binding activity [11, 12], whereas ORF2-encodes a protein (ORF2p) with demonstrated reverse transcriptase (RT) [13] and endonuclease (EN) activities [14]. Both proteins are required for retrotransposition in *cis* [15]. Notably, along with mobilizing its own RNA, L1 activity is responsible for dispersing eight thousand processed pseudogene insertions [16–20], more than 1.2 million Alu elements – a type of SINE – and ~2700 SINE-R/VNTR/Alu (SVA) elements throughout the human genome [7, 21–25].

Although ORF1p is critical for retrotransposition its roles in *cis* and *trans*-mobilization are incomplete [26]. While human ORF1p does not display amino acid sequence similarity to other known proteins [27]; recent studies have revealed that the 40 kDa ORF1p has three distinct domains: coiled-coil (CC), RNA recognition motif (RRM) and carboxy terminal domain (CTD) [28, 29]. In-vitro studies have demonstrated that both human and mouse ORF1p are non-sequence specific single stranded RNA and DNA binding proteins with nucleic acid chaperone activity [12, 28, 29]. Studies on the localization of these proteins in cell lines suggest that ORF1p is mainly cytoplasmic and may concentrate in certain regions of the cytoplasm resulting in the formation of cytoplasmic foci [30–34]. Interestingly, a fraction of ORF1p is observed in stress granules and in the nucleus of cells [32, 33, 35]. Furthermore, detection of ORF1p and its cytoplasmic and nuclear localisation has also been reported in healthy and cancer human tissues [36–38].

Although L1 retrotransposition in most somatic cells is generally silenced by a variety of defence mechanisms and host factors, such as the APOBEC3 proteins, [39–42] presumably to limit insertional mutagenesis, transgenic animal models and deep-sequencing studies have shown that L1 is highly active in certain regions of the brain (e.g. *hippocampus*) [2, 43–45]. The pioneering work of Muotri et al. [2], which involved insertion analysis of transgenic mice carrying an engineered L1 that upon retrotransposition expresses green fluorescent protein (GFP) [46], unexpectedly identified retrotransposition-competent cells in many regions of the brain including cortex, hypothalamus, cerebellum and hippocampus. While an increase in L1 copy-number has been observed using qPCR or next-generation sequencing in certain brain disorders like ATM deficiency [47, 48], Rett Syndrome, schizophrenia [49] and autism [50], the biology of L1 proteins in the human brain of healthy individuals that were not diagnosed with any neurodegenerative disease is poorly understood.

Here, we describe a novel polyclonal antibody against the RRM domain of human L1-ORF1p which we generated to investigate L1 protein expression in the human brain. Using this antibody, we detect ORF1p in various parts of the human brain derived from post-mortem samples. Interestingly, we observe differential expression of ORF1p when comparing the same brain region of samples from different ages. Together, these data provide in vivo evidence for L1 protein expression in the human brain and describe a new antibody available to the community.

## Methods

### Cloning and purification of RRM domain from L1-ORF1p and generation of polyclonal antibody against human L1-ORF1p (RRM)

**Fig. 1:**
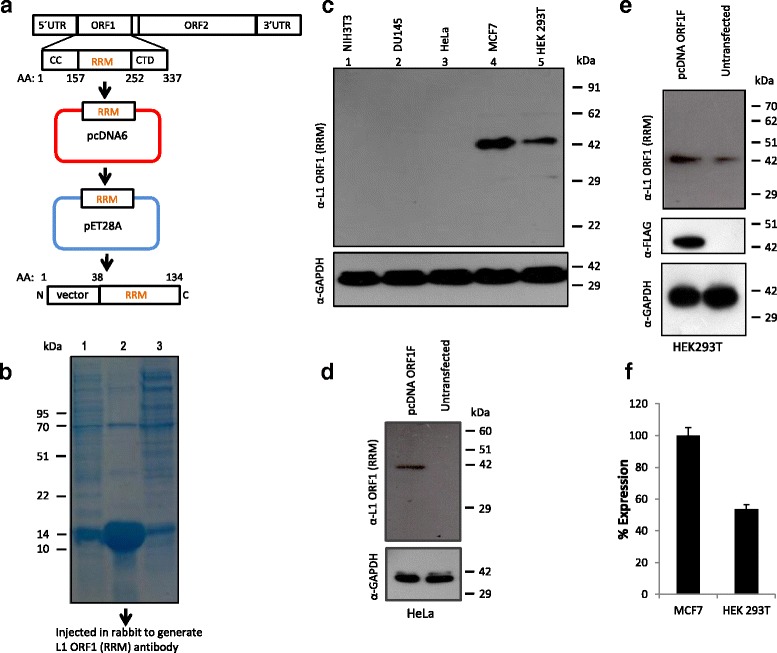
Generation and characterization of α-human L1 ORF1p (RRM) antibody: **a** Diagram of full-length active human L1 retrotransposon. L1 encodes two proteins (ORF1p and ORF2p). ORF1p is characterized by three distinct domains: coiled-coil (CC), RNA Recognition Motif (RRM) and Carboxy Terminal Domain (CTD). The RRM domain alone (amino acids 157–252) was sub-cloned in pET bacterial expression vector. **b** SDS-PAGE of *E.coli* expressed pET human L1 ORF1p (RRM) peptide. Lane 1: soluble fraction, Lane 2: elution, Lane 3: flow through. A contaminate protein (less than 1% of the total amount) with molecular mass of around 70 kDA was also eluted with RRM domain. The eluted ORF1p (RRM) peptide was injected into a rabbit for generating α-hORF1p (RRM) antibody. **c** Immunoblot analysis of cell lysates from diverse murine and human cell lines using the α-hORF1p (RRM) antibody.Lane1: NIH3T3 (mouse embryonic fibroblast), Lane 2: DU145 (human prostate cancer cell line), Lane 3: HeLa (human epithelial cancer), Lane 4: MCF-7 (human breast cancer) and Lane 5: HEK293T (human embryonic kidney). Panel 2- immunoblot with α-GAPDH (loading control). **d** Immunoblot analysis of L1 ORF1p expression in pcDNA-ORF1F-transfected HeLa cells. Using the α-hORF1p antibody, ORF1p expression is detected in transfected HeLa cells but not detectable in untransfected HeLa cells. (Construct: ORF1F; ORF1 tagged with C-terminal FLAG cloned in pcDNA vector); Panel 2- immunoblot with α-GAPDH (loading control). **e** Immunoblot analysis of ORF1p expression in pcDNA-ORF1F-transfected HEK 293 T cells. Lane 1: transfected, Lane 2: Untransfected. Panel 1: immunoblot with α-human L1 ORF1p (RRM), Panel 2: with α-FLAG and Panel 3: with α-GAPDH (loading control) **f** Quantification of ORF1p amount by image J software (https://imagej.net/ImageJ) in total lysate in from MCF-7 and HEK 293 T cells

The RNA Recognition Motif (RRM) domain of human ORF1 from L1-RP (Accession number -AF148856.1) [51] was selected (Fig. 1a and Additional file 1) as the epitope to immunize a rabbit for antibody generation. The RRM domain was isolated from ORF1-RRMF (RRM domain cloned at *EcoRI-NotI* sites of pcDNA6/myc-HisB) [20, 52] using *EcoRI* and *NotI*. The ORF1-RRM fragment was cloned into *EcoRI* and *NotI* of pET-28a vector (EMD Biosciences) for protein expression in bacteria. The expressed protein and corresponding nucleotide sequence are provided in Additional file 1. The His-tagged L1-ORF1-RRM protein was expressed in *E. coli* strain BL21 and purified on nickel-NTA Agarose (Qiagen) according to the manufacturer’s protocol. Purified human ORF1 RRM domain, with molecular mass of approximately 15 kDa (vector sequence plus RRM, details in Additional file 1), was used to immunize rabbit (Immunization protocol: Additional file 1). Immunized whole serum from the rabbit without further purification was used for the experiments described in this study to detect ORF1p in cell and tissue lysates.

### Cell culture and Transfection

HEK293T (human embryonic kidney), HeLa (cervical carcinoma), MCF-7 (Breast cancer), DU145 (Prostate cancer), and NIH3T3 (Mouse fibroblast) cells were maintained in a tissue culture incubator at 5% CO_2_, 37 °C in high glucose Dulbecco’s modified Eagle medium (DMEM) supplemented with 10% fetal bovine calf serum (Gibco, Thermo Fisher Scientific) and 100 U/ml penicillin-streptomycin (Gibco, Thermo Fisher Scientific). For transfections, cell lines were seeded into a 35 mm plate to achieve 30–50% confluency within 8–12 h prior to transfection. Using Fugene 6 (Promega), 1–1.5 μg of plasmid DNA was transfected into the cell lines according to manufacturer’s instructions. Transfected cells were incubated for an additional 48 h before proceeding to any experiment.

#### Protein extraction and immunoblots

Whole cell lysates from cell lines were prepared using lysis buffer A [composition: 20 mM Tris-Cl pH 7.8‚137 mM NaCl and 1% NP-40 supplemented with 1X protease inhibitor cocktail (Roche)]. The lysate was cleared by centrifugation at 2500×g, 5 min at 4 °C. To prepare brain tissue lysate, around 150–200 mg frozen brain tissue (post mortem frontal cortex tissue from 80 year old) was crushed in mortar pestle using liquid nitrogen and transferred to 1.5 ml tube containing 250 μl cold RIPA buffer [150 mM NaCl, 1% NP-40, 0.5% Na-deoxycholate, 0.1% SDS, 50 mM Tris-Cl pH -8.0 with protease inhibitor cocktail (Roche)]. The crushed tissue was then passed through an 18 gauge needle 5–8 times and incubated on ice for 45 min with intermittent mixing. Finally, the lysate was cleared by centrifugation (12,000×g, 10 min, 4 °C) and supernatant transferred to a new tube and stored at −80 °C until further use. The Bradford reagent (Bio-Rad) was used to estimate the protein concentration. The proteins were separated by SDS-PAGE (Mini protein Tetra cell Bio-Rad) and wet transferred to nitrocellulose membrane (Millipore) by applying 100 V for 75 min (Bio-Rad mini trans blot electrophoretic transfer cell). The protein was detected by Western blot using the following primary antibody: polyclonal rabbit anti human L1 ORF1 (1:33,000, and 1:20,000 dilution), anti-GAPDH (1:6000 dilution) (Santa Cruz Biotechnology, anti-FLAG (1,3000 dilution) (Sigma). Secondary anti- rabbit HRP and secondary anti-mouse HRP were purchased from Jackson ImmunoResearch Laboratories, USA. Western blots were developed using ECL western blotting detection reagent (Pierce) as per manufacturer’s instructions.

#### Immunofluorescence analysis

2 × 10^5^HeLa and MCF-7 cells were seeded on sterile Poly-L- lysine coated cover slips in 35 mm tissue culture plates 12–18 h prior transfection. The following day, cells were transfected using 1 μg of plasmid DNA (prepared using GeneJet Plasmid miniprep Kit, Thermo Scientific) and 3 μl of Fugene 6 Transfection Reagent. The immunofluorescence protocol was adapted from “Abcam protocol” (http://www.abcam.com/protocols/immunocytochemistry-immunofluorescence-protocol) with minor modifications.One day post-transfection, media was aspirated, cells were washed with ice cold 1XPBS and fixed by incubating the cells in 100% chilled (−20 °C) methanol for 10 min. at room temperature. Next, fixed cells were washed three times for 10 min with immunofluorescence wash buffer (composition: 0.05% sodium azide, 0.1% BSA, 0.75% glycine, 0.04% Tween-20, and 0.2% Triton X-100 in 1X PBS) using gentle agitation. Permeabilization of fixed cells was performed by incubating cells in permeabilization buffer (1X PBS containing 0.5% Triton X-10) for 3–5 min. at room temperature. Afterwards, cells were rinsed with immunofluorescence wash buffer three times and each time cells were allowed to sit in the wash buffer for 5 min for better quenching. The fixed and permeabilized cells were blocked for an hour in room temperature by incubating in blocking solution (1% BSA in 1XPBST). Subsequently, cells were incubated with human α-ORF1p (RRM) primary antibody (1:500 diluted in blocking solution) at 4 °C overnight. The next day, cells were washed three times with immunofluorescence wash buffer as stated above followed by incubation with secondary antibody [Alexa fluor 488; Jackson Immuno Research laboratories (1:300 diluted in blocking solution)] for one hour at room temperature in a dark room. Immediately after this, cells were rinsed twice in immune fluorescence wash buffer for five minutes at room temperature. After washing, cells were counterstained with Hochst 33342 for 10 min at room temperature and mounted on slides with DPX mounting media. Samples were then analysed with appropriate fluorescent filters on confocal laser scanning microscope (LSM 780, Carl Zeiss, Germany).

**Tissue Specimens:** Brain tissue samples were collected in the form of formalin-fixed paraffin embedded (FFPE) sections on slides and frozen tissue from the Human Tissue Repository for Neurobiological Studies (HBTR), Human Brain Bank, Department of Neuropathology, National Institute of Mental Health and Neurosciences, (Bangalore, India). Following proper consent, all the samples were collected from victims of road traffic accident. The tissues were taken from zones distal from the site of injury. All investigations were conducted in accordance with ethical principles embodied in the declaration of tissue request and material transfer agreement [IHEC No. BT/IHEC-IITR/2017/6673; Institute Human Ethics Committee (IHEC), Indian Institute of Technology Roorkee, Utarakhand, India].

#### Immunohistochemistry (IHC)

Paraffin-embedded brain tissue sections on glass slides were de-paraffinized rehydrated in descending grade of ethanol solutions before proceeding for antigen retrieval. The antigen retrieval step was adapted from “Abcam protocol” (http://www.abcam.com/protocols/immunocytochemistry-immunofluorescence-protocol). The process was performed in a common household vegetable steamer (pressure cooker) using Tris-EDTA antigen retrieval buffer (10 mM Tris base, 1 mM EDTA solution, 0.05% Tween 20, pH -9.0). Next, slides were washed 2 X 5 min each in TBST (1X TBS containing 0.025% Triton-X100) and then blocked in blocking solution (1% BSA in 1X TBST) for 1 h at room temperature. Thereafter, slides were incubated with polyclonal rabbit α-ORF1p (RRM) antibody (1:500 diluted in blocking reagent) at 4 °C overnight in humid chamber. The next day, slides were washed with 1X TBST and treated with 0.3% hydrogen peroxide to quench any peroxidise present within the tissue. Slides were then incubated with secondary antibody [Goat α-Rabbit HRP 1: 500 dilution (Jackson ImmunoResearch)] for an hour at room temperature. The slides were washed 3 × 10 min at room temperature with gentle agitation. Signals were visualised by adding 3–3’- Dia amino benzidinetetrahydrochloride (DAB substrate) solution to the slides and were counterstained with haematoxylin, (Himedia) dehydrated with ascending order of ethanol and mounted with DPX mounting media. Images were captured using a light microscope (Leica Microsystems) equipped with a camera. Intensity of DAB stained regions was measured with ImageRatio software [53] and plotted as a percentage of expression. α-Neurofilament (NE14) (Biogenex) raised in mouse was used as neuronal marker (1:500 dilution) that preferentially stained the neurons.

## Results

### Characterization α-human ORF1p (RRM) antibody by immunoblotting and immunofluorescence

Human L1 ORF1p is a 338 amino acids (L1RP, Accession number: AF148856.1) protein with a predicted mass of 40 kDa [11, 51] with RNA binding and nucleic acid chaperone activity [12].ORF1p is characterized by three distinct domains: Coiled Coil (CC) (AA: 52–153 relative to L1RP Accession number AF148856.1), RNA Recognition Motif (RRM) (AA: 157–252) and Carboxy Terminal Domain (CTD) (AA: 264–323) (Fig. 1a) [29]. Although much has been learned from cell culture and genomic studies about L1 biology, our understanding of retrotransposition in vivo is far from complete. Here we sought to generate an additional tool to investigate L1 activity, namely an antibody reactive to ORF1p that would be useful for detecting the native protein. To generate ORF1p antibody we selected the RRM domain as the epitope of interest for three reasons: 1) a previous study [29] showed high expression of this domain, 2) the same study showed that the expressed protein was retained in the soluble fraction (native form) in a bacterial expression system and 3) the RRM domain is easier to handle due to its smaller size (MW 12 kDa) relative to full-length ORF1p (MW 40 kDa). The RRM domain from human (h) ORF1 was cloned into a bacterial expression vector (Fig. 1a and Additional file 1: Figure S1). Following expression in bacteria, the RRM domain was purified using Ni- agarose chromatography. Analysis of the purified protein by SDS-PAGE and Coomassie staining revealed a distinct band of ~15 kDa and a minor contaminant protein (less than 1% compared to the RRM band) at ~70 kDa (Fig. 1b).

To generate a hORF1p specific antibody, we injected the purified RRM domain into a New Zealand rabbit. Following isolation, serum was assayed for α-hORF1p (RRM) by Western blot analysis on protein lysates generated from human cancer cell lines known to express varying levels of ORF1p (U87, MCF7, HeLa, Du145 and HEK293T) (Fig. 1c). Robust expression of a 40 kDa protein- approximate mass of ORF1p- was detected in MCF-7 and HEK293T cell lines [20, 38, 54] while DU145 and HeLa cells lacked detectable expression (Fig. 1c). Loading the same amount of total protein lysate followed by western blot analysis using increased serum concentration (1:20,000 instead of 1:33,000 dilution) (Additional file 1: Figure S2a) revealed an extra band of lower molecular weight (~25 kDa) only in samples containing the 40 kDa band. To assess sensitivity of α-hORF1p (RRM), we carried out western blot analysis using increasing amounts of total lysate from HEK293T cells (10 μg, 20 μg and 40 μg) which revealed a distinct single band at 40 kDa when the primary antibody was used at a 1:33,000 dilution (Additional file 1:Figure S2b, Panel 2); while a similar experiment with the same amount of protein lysate but more concentrated serum (1: 20,000) detected a smaller ~25 kDa band in the lane loaded with 40 μg and 20 μg of protein lysate (Additional file 1: Figure S2b, Panel 1). Furthermore, we performed western analysis using total lysate from the *E.coli* expression cells (pET30b induced in *E.coli* BL-21 strain). The absence of other non-specific bands suggested that the small fraction (less than 1% of RRM band) of unknown 70 kDa bacterial protein which co-purified with the RRM peptide was not immunogenic in rabbit (Additional file 1: Figure S2c).

Quantification of band intensity by densitometry revealed that ORF1p expression in MCF-7 cells is almost twice the amount detected in HEK293T cells (Fig. 1C, lane 4 and lane 5; Fig. 1f).Consistent with species-specificity, serum failed to detect any band in cell lysate obtained from a mouse cell line [Fig. 1C, lane 1 (NIH-3T3)]. To further characterize specificity, we assayed reactivity of serum on protein lysates from HeLa and HEK293T cells transfected with a construct containing L1-ORF1 sequence tagged by a FLAG-epitope at the C-terminus of ORF1 (pcDNA-ORF1F) [20] (Fig. 1d, Panel 1), (Fig. 1e, Panel 1). α-FLAG and α-GAPDH served as controls (Fig. 1d, Panel 2), (Fig. 1e, Panel 2 and Panel 3). Along with demonstrating that the serum isolated from the rabbit injected with the hORF1p (RRM) peptide contains an antibody reactive and specific to human L1-ORF1p, these data indicate that our antibody is capable of detecting endogenous denatured L1 protein.

**Fig. 2:**
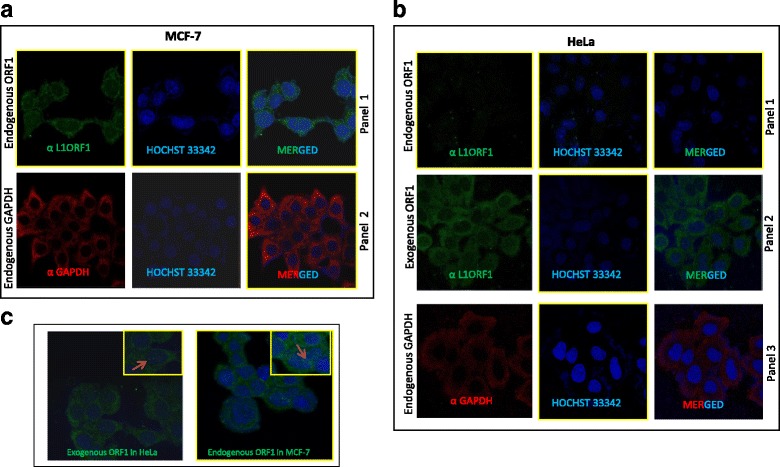
Immunofluorescence analysis reveals that α-human L1 ORF1p (RRM) can detect over expressed and endogenous ORF1p in its native conformation. **a** Panel 1: Endogenous ORF1p (green) detection in MCF-7 cells using α-human L1 ORF1p (RRM). Hochst (blue) was used to stain nuclear DNA. A merged image is shown (top right). Panel 2: Endogenous GAPDH protein served as a control of IHC technique. **b** Panel 1: Detection of endogenous ORF1p in HeLa cells (Left column).Nuclear DNA was stained with Hochst (middle column). Merged image shown in (top-right). Panel 2: Detection of exogenous ORF1 (green) in HeLa cells after transfecting pcDNA ORF1F [ORF1 green (left column), Hochst blue (middle column), merged (rightmost column)]. Panel 3: Endogenous GAPDH expression in HeLa cells. **c** Mostly cytoplasmic localization of ORF1p in MCF-7 (endogenous) and HeLa (exogenous) cells; however, some cells display nuclear localization of ORF1 protein (indicated by arrow)

To determine whether α-hORF1p (RRM) can detect endogenous hORF1p in its native conformation, we performed immunofluorescence (IF) on cultured MCF-7 and HeLa cells characterized for the presence or absence of L1 ORF1p by immunoblot analyses (Fig. 2). Consistent with our Western blot data, no fluorescence was detected in untransfected HeLa cells (Fig. 2b, Panel 1); however, transfection with pcDNA-ORF1F revealed cytoplasmic staining (Fig. 2b, Panel 2). Indeed, cytoplasmic localization of exogenous ORF1p has been reported for a variety of cell lines including HeLa [31, 33, 35]. These data indicate that α-hORF1p (RRM) can detect over-expressed ORF1p in its native conformation in fixed HeLa cells by IF.

To assay whether α-hORF1p (RRM) can detect endogenous hORF1p, we carried out IF on MCF-7 cells (Fig. 2a). In agreement with our hypothesis, we detected endogenous ORF1p using α-hORF1p (RRM) (Fig. 2a) and the localization mirrored that of the exogenously transfected ORF1p in HeLa cells (Fig. 2b); specifically, we observed mainly cytoplasmic staining of ORF1p in MCF-7 cells by immunofluorescence (Fig. 2a, Panel 1). GAPDH serves as an internal control (Fig. 2a, Panel 2). Notably, few cells (less than 5%) both in MCF-7 (endogenous ORF1p) and HeLa (exogenous ORF1p) showed nuclear localisation of ORF1p (Fig. 2c). The data demonstrate that α-hORF1p (RRM) is able to detect endogenous ORF1p in a cancer cell line (Fig. 1c and 2a) and that minimal background fluorescence is observed in cell lines lacking ORF1p expression by Western blot analysis using our antibody (Fig. 2b).

### Detection of endogenous ORF1p in human tissues using α-hORF1p (RRM)

While significant progress has been made recently regarding our understanding of endogenous L1 activity primarily using next-generation sequencing technology for insertion analysis in disease states [49, 50] and animal models [2] such as mouse, less is known about human L1 retrotransposition and protein functions in vivo in somatic tissues. To this end, we carried out immunohistochemistry using α-hORF1p (RRM) on a variety of human samples, including brain tissues [36, 38] where L1 insertional activity is known to be increased [2, 43, 44].

**Fig. 3:**
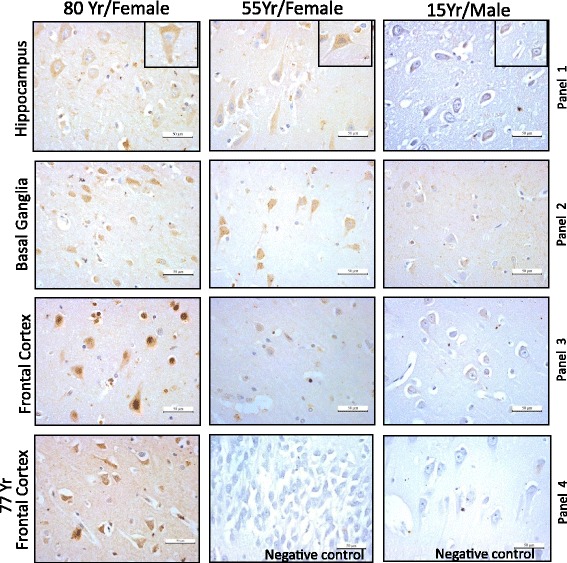
Immunoperoxidase detection of endogenous L1-ORF1p in different regions of sections of the normal human brain. Human α-L1 ORF1p (RRM) was used to detect the expression L1 activity in human three different brain tissues: hippocampus, basal ganglia and frontal cortex. Samples from three different aged brains (80 year, 55 year and 15 year old) were analyzed. Images were taken at 40X magnifications. Panel 4, leftmost: Immunoperoxidase staining of frontal cortex from 77 year old individual. Panel 4 middle: As a negative control, the immunostaining procedure was performed without primary antibody on a hippocampus section from a 55-year old individual. Panel 4 rightmost: In another negative control, IHC was performed on the same section using primary α-hORF1p (RRM) (raised in rabbit) followed by secondary α-mouse

To assay for ORF1p expression in different regions of human brain, we performed immunohistochemistry (IHC) on formalin fixed paraffin embedded brain sections. We first examined L1-ORF1p expression in three different regions - frontal cortex, hippocampus and basal ganglia of a brain from a post-mortem 55 years old female (victim of a traffic accident) with no known neurological or psychiatric illness. All three regions show significant staining in neurons with α-hORF1p (RRM) (Fig. 3). A hippocampus section exposed to the secondary antibody alone (Fig. 3, panel 4 middle) did not exhibit any specific immunostaining (negative control).To assay specificity of α-hORF1p (RRM) in IHC, we utilized an additional three controls: 1) primary α-hORF1p (RRM) (raised in rabbit) followed by secondary α-mouse (Fig. 3, Panel 4 rightmost), 2) primary α-His (raised in mouse) and secondary α-mouse (Additional file 1: Figure S2d) and 3) primary non-immune sera (rabbit) and secondary α-rabbit (Additional file 1: Figure S2e); in all the instances, no signal was detected. In another control, total lysate from 80 year old frontal cortex tissue and MCF-7 cells probed with non-immune rabbit sera by immunoblotting didn’t show any signal (Additional file 1: Figure S2f). To determine whether the cells which stained with α-hORF1p (RRM) are neurons, we performed IHC using α-Neurofilament (NE-14) a neuronal marker in a hippocampus section from 55 year-old brain (Additional file 1: Figure S3b). These data demonstrate that NE-14 stained neurons show morphological similarities with α-hORF1p (RRM) staining cells. To account for age or sex bias potentially associated with L1-ORF1p expression, we stained post-mortem samples from a 15-year-old male and an 80-year-old female. These data show that ORF1p levels are noticeably lower for the 15-year-old sample across all three regions tested (frontal cortex, hippocampus and basal ganglia) when compared to stained samples from the 55-year-old and 80- year-old individuals (Fig. 3). Surprisingly, we observed very high ORF1p expression in the 80-year-old frontal cortex (Fig. 3, Panel 3 leftmost). Staining another frontal cortex section from 77-year-old brain showed similar very high expression of ORF1p (Fig. 3, Panel 4 leftmost). Quantification of DAB signal (e.g. ORF1p positive cells) using ImmunoRatio software [53] indicate that ORF1p expression in the hippocampus and basal ganglia samples from the 55 -year-old and 88-year-old individuals is similar (Additional file 1: Figure S3a, Panel 1 and Panel 3). In contrast, ORF1p expression in samples from the 80-year-old frontal cortex (Fig. 3, Panel 3 leftmost; Additional file 1: Figure S3A, Panel 3) are approximately 3-fold more intense relative to the frontal cortex sample from the 55-year-old. Furthermore, the expression of ORF1p observed in the 15-year-old frontal cortex is less than 5% of that observed in the sample from the 80-year-old (Additional file 1: Figure S3a, Panel 3).

While quantification of ORF1p expression in the basal ganglia samples from the three individuals showed similar levels (Additional file 1: Figure S3, Panel 2), the signal intensity of stained cells coming from the 15-year-old individual (Fig. 3, Panel 2 rightmost) was significantly less. We speculate that the increased value for the basal ganglia for the 15-year-old is due to increased tissue matrix staining, a technical problem we were unable to circumvent.

**Fig. 4:**
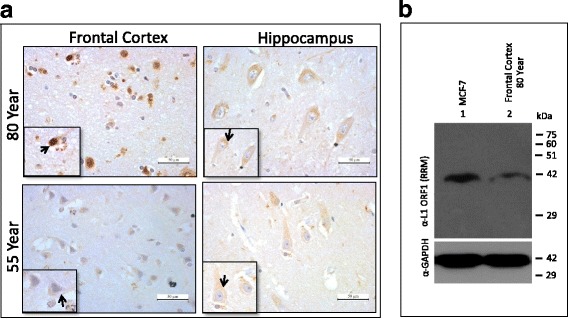
Nuclear-cytoplasmic localization of endogenous ORF1p in frontal cortex and hippocampal sections obtained from 55 year- and 80 year-old brain. **a** Differences in the abundance of nuclear-localized ORF1p between frontal cortex relative to hippocampal section from 80 year-old. Immunohistochemistry analysis was carried as described in methods using α-L1 ORF1p (RRM). **b** Detection of L1 ORF1p in total lysate from 80 year old frontal cortex by Western blotting. α-human L1 ORF1p (RRM) detects ORF1p (~40 kDa) in total lysate from 80 year old frontal cortex (lane 2). Total lysate from MCF-7 cells was used as control (lane 1)

Along with gaining insight into the tissue distribution and abundance of L1-ORF1p, IHC can provide insights regarding the sub-cellular distribution of ORF1p. Similar to our IF analysis (Fig. 2), we observe ORF1p primarily in the cytoplasm of all three brain regions. Interestingly, the frontal cortex of the 80-year-old showed intense staining of ORF1p in the nucleus; this pattern was not observed for the other regions or other samples tested (Fig. 4a). To complement ORF1p detection in different parts of post-mortem human brain section by IHC, we performed Western blot analysis using total lysate from the 80 year old frontal cortex. Using total lysate from MCF-7 cells as a control, we were able to detect ORF1p in the 80 year old frontal cortex tissue (Fig. 4b, Panel 1). The GAPDH immunoblotting was used as an internal control (Fig. 4b, Panel 2). These data further support that L1 ORF1p is present in different anatomical regions of human brain including robust levels in the frontal cortex region.

**Fig. 5:**
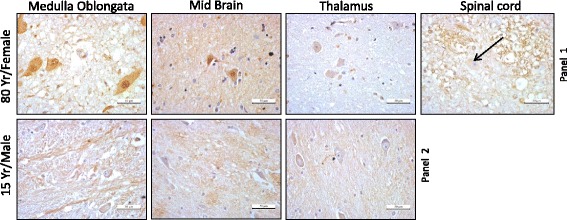
Immunoperoxiadse detection of endogenous ORF1p in medulla oblongata, midbrain, thalamus and spinal cord in sections from 80 year female and 15 year male

To further our interrogation of ORF1p expression in the human brain, we carried out IHC on additional sections derived from the following regions: medulla oblongata, midbrain, thalamus and spinal cord (Fig. 5). Notably, we did not have access to any of these tissues in the case of the 55-years old individual, and there was no spinal cord tissue available from the 15-years old. For tissues derived from the 80-year-old individual, we detected ORF1p positive cells for the medulla oblongata, midbrain and thalamus but not the spinal cord (Fig. 5, Panel 1 rightmost). Consistent with barely detectable amounts of L1 ORF1p in the 15-year-old individual’s (Fig. 3) frontal cortex, basal ganglia and hippocampus, our IHC experiments did not provide any evidence for the presence of L1 ORF1p in thalamus, midbrain, and medulla oblongata of the same individual (Fig. 5, Panel 2).

**Fig. 6:**
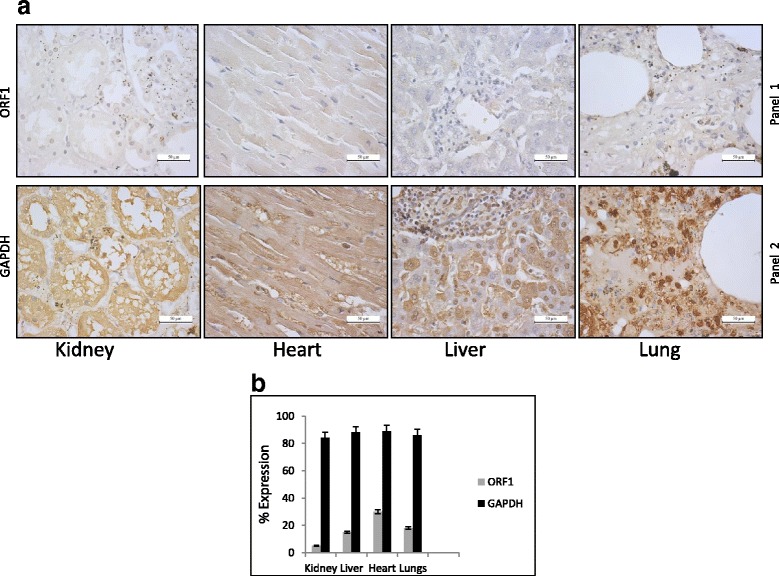
Limited expression of endogenous ORF1p in non-brain tissue sections. **a** Detection of ORF1p using α-L1 ORF1p (RRM) by immunohistochemistry in kidney, heart, liver and lung. GAPDH staining functions as a control. **b** Quantification of ORF1p and GAPDH in kidney, heart, liver and lung using ImmunoRatio software [53]

In addition to the brain, L1 may also be expressed in other somatic tissues in vivo although at least one other study suggests otherwise [38]. To test if L1 is expressed in other human tissues besides the brain, we assayed three tissues previously tested for ORF1p expression by IHC [38] kidney, liver and lung along with heart tissues using α-hORF1p (RRM). Consistent with Rodic et al. data, our immunohistochemistry analysis also suggests little to no ORF1p expression in these tissues (Fig. 6a, Panel 1). IHC for GAPDH expression showed very high expression in all four tissues tested (Fig. 6a, Panel 2) as well as in brain sections (data not shown). Quantitation using ImmunoRatio software [53] showed GAPDH expression is comparable in all four tissues whereas expression of ORF1p is more abundant in heart tissue compared to other non-brain samples (Fig. 6b).

## Discussion

### ORF1 protein expression in the brain is widespread

Recently, the role of retrotransposon activity in brain function and neuronal plasticity has gained significant interest. Although several studies to date have reported an increase in L1 insertions in certain brain regions such as the hippocampus [2, 43, 45], our understanding of L1 protein expression in the brain is limited. In this study, we report the first in vivo detection of L1 protein expression in sections from multiple distinct regions of the post-mortem human brain. (Figs. 3, 4, and 5).

ORF1p is one of two proteins encoded by LINE-1 retrotransposons; both of which are required for retrotransposition *in cis* [15]. While significant insights pertaining to ORF1p biology have been gained from cell culture, biochemical, genetic, and structural studies, less is known regarding its function in vivo. Here, we set out to establish a new reagent - α-hORF1p (RRM) – that would be useful to complement existing tools and studies [24, 37, 38, 54, 55] including insertion analysis in brain tissues using next-gen sequencing.

Using four different cancer cell lines (MCF-7, HeLa, DU145, HEK293T) we observe very high levels of endogenous ORF1p in MCF-7 and HEK293T cells, but are unable to detect ORF1p in lysates from HeLa and DU145 cells by Western analysis. These data are consistent with previous studies which have also observed high levels of endogenous ORF1p in many breast cancer tumors [37] and breast cancer cell lines (T47D, SKBr3, BT-20, MCF-7, Hs578T) [54]. Even though L1 protein expression and the retrotransposition-competence of a cell (e.g., new insertions) is known to vary across cancers and cancer cell lines [38, 56–58] perhaps MCF-7 cells might be useful in identifying factors important for ORF1p expression and stability.

### A potential association between LINE-1 ORF1p expression and aging

The detection of ORF1p by immunohistochemistry and its quantification in several distinct regions of the human brain derived from different individuals provides additional support that L1 is indeed active in this organ. We observe differences in the intensity and abundance of ORF1p in the brain tissue samples across individuals. Specifically, tissues derived from two individuals older than 50-years of age displayed markedly increased levels of ORF1p relative to samples derived from a 15-year-old male (Fig. 3). These data are supported by previous studies which have identified variation in L1 copy number using qPCR-based assays across different brain regions and individuals; but, the exact relationship between endogenous L1 protein expression and insertion frequency remains incomplete [43]. Importantly, the data here assaying seven different brain regions (frontal cortex, hippocampus and basal ganglia, thalamus, midbrain, medulla oblongata and spinal cord) along with four other organs (liver, lung, kidney and heart) from three different individuals increase our understanding of human tissues that permit endogenous ORF1p expression.

Consistent with our detection of robust endogenous ORF1p expression in the hippocampus and frontal cortex, an elevated insertion frequency has been observed in these tissues by single-cell analysis and deep-sequencing [45]. Our quantification of staining indicates significantly higher expression of ORF1p in basal ganglia, hippocampus and frontal cortex when compared to other brain regions tested in samples derived from the 55-year old. Interestingly, our data indicate that samples originating from even older brains (e.g. 77- and 80-years-old) display more detectable expression of ORF1p. Perhaps the most striking finding from IHC analysis of brain tissues is the near absence of ORF1p staining in samples derived from a 15-year-old brain in light of our ability to easily detect ORF1p in samples from older brains.

Importantly, the frequency and impact of new insertions in brain tissues is still being debated. For instance, single neuron sequencing performed by [59] to assay rates of retrotransposition in the frontal cortex and caudate nucleus calculated less than 0.1 insertions per neuron. Their data suggested that an increase in ORF1p expression within a particular region of the brain might serve some other function and does not correlate with the number of L1 insertions in that region of the brain. Consistently, our data assessing ORF1p expression in non-brain tissues like kidney, heart, liver and lung did not detect expression of ORF1p in agreement with Rodic et al. [38]. In contrast, IHC analysis of adult testis, another non-brain tissue have demonstrated significant expression of ORF1p and ORF2p by IHC analysis [36].

Although we acknowledge that the sample size is very small in this study, it is tempting to speculate that endogenous ORF1p expression increases with age. However, at this time we cannot rule out that the observed ORF1p expression differences seen in this study may be due to inter-individual variation in the number of “hot” L1s each person inherits [60]. Relatedly, the longevity regulating protein Sirtuin 6 (SIRT6) has been reported to suppress L1 retrotransposition. Specifically, SIRT6 enforces silencing of L1 by establishing transcriptionally repressive heterochromatin at L1 genomic sequences [61]. With aging, SIRT6 activity is depleted allowing the activation of silenced L1 elements [61]. Future studies similar to this report, which include ORF2p reverse transcriptase assays and deep-sequencing analysis for the genomic L1 insertion content, will likely resolve whether older brains display elevated rates of L1 retrotransposition (e.g., an increase in L1 insertions) relative to younger brains and any associated biological impact.

## Conclusions

Our findings show elevated expression of L1ORF1p in different parts of post-mortem human brain compared to other body parts like kidney, heart, liver and lung. We have seen individuals of different ages display very different expression of L1ORF1p, especially in the frontal cortex. Overall, our data show ORF1p levels in brain tissues vary from person to person where age might have some influence on L1 retrotransposition.

## Additional files

Additional file 1: Supplementary text and Figures S1-S3. (ZIP 1116 kb)

## Abbreviation

BSA: Bovine Serum Albumin
CC: Coiled Coil
CTD: Carboxy Terminal Domain
DAB: 3–3′- Diaaminobenzidinetetrahydrochloride (DAB substrate)
DMEM: Dulbecco’s Modified Eagle medium
DNA: Deoxyribonucleic Acid
ECL: Enhanced Chemiluminescence
EDTA: Ethylene-Diamine Tetra-Acetic Acid
FFPE: Formalin-Fixed Paraffin Embedded
GAPDH: Glyceraldehyde 3-Phosphate Dehydrogenase
HBTR: Human Brain Tissue Repository
HEK: Human Embryonic Kidney
HRP: Horseradish Peroxidase
IF: Immunofluorescence
IHC): Immunohistochemistry
kDa: kilo Dalton
LINE: Long INterpersed Element
MCF-7: Michigan Cancer Foundation-7
mm: Millimeter
mM: Millimolar
MW: Molecular Weight
NaCl: Sodium Chloride
^o^C: degree centigrade
ORF: Open Reading Frame
PBS: Phosphate Buffered Saline
PBS-T: Phosphate Buffered Saline-Tween
qPCR: quantitative Polymerase Chain Reaction
RNA: Ribonucleic Acid
RRM: RNA Recognition Motif
SDS-PAGE: Sodium Dodecyl Sulphate-Polyacrylamide Gel Electrophoresis
SINE: Short Interpersed Element
SIRT6: Sirtuin 6
SVA: (SINE-R/VNTR/Alu)
TBST: Tris Buffered Saline-Tween
TBST: Tris Buffered Saline-Tween
UTR: Untranslated region
V: Volt
x g: Times gravity
μg: microgram
