# Supplementary material for: Detection of the LINE-1 retrotransposon RNA-binding protein ORF1p in different anatomical regions of the human brain
Source: Mob DNA. 2017 Nov 22;8:17. doi: 10.1186/s13100-017-0101-4 (PMC5700708; doi:10.1186/s13100-017-0101-4)
Supplement: Supplementary file 1 — Supplementary text and Figures S1-S3. (ZIP 1116 kb) [file 13100_2017_101_MOESM1_ESM.zip › Supple Figures _Sur et al RRsub.pptx]

## Slide 1
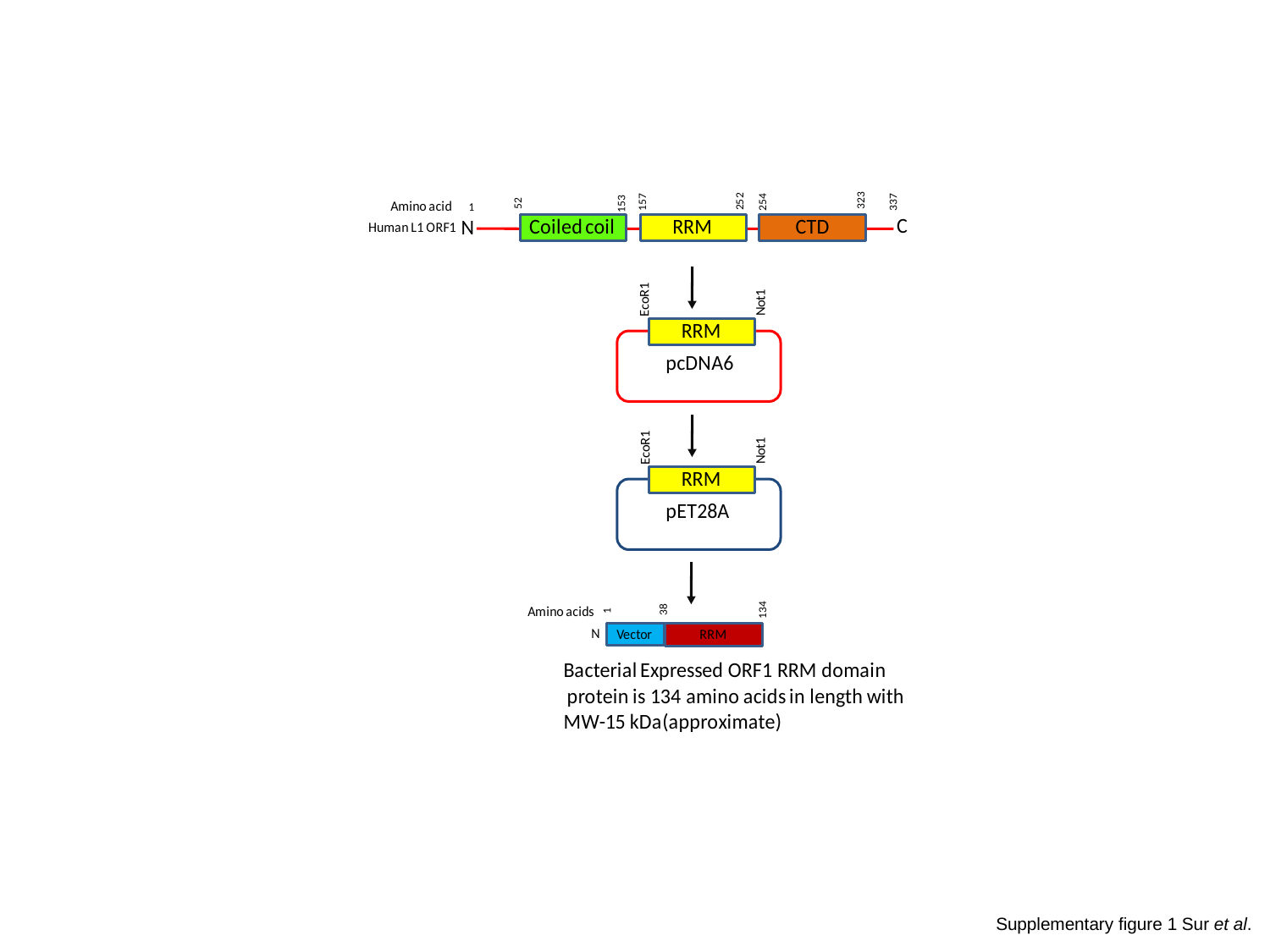

Supplementary figure 1 Sur et al.

## Slide 2
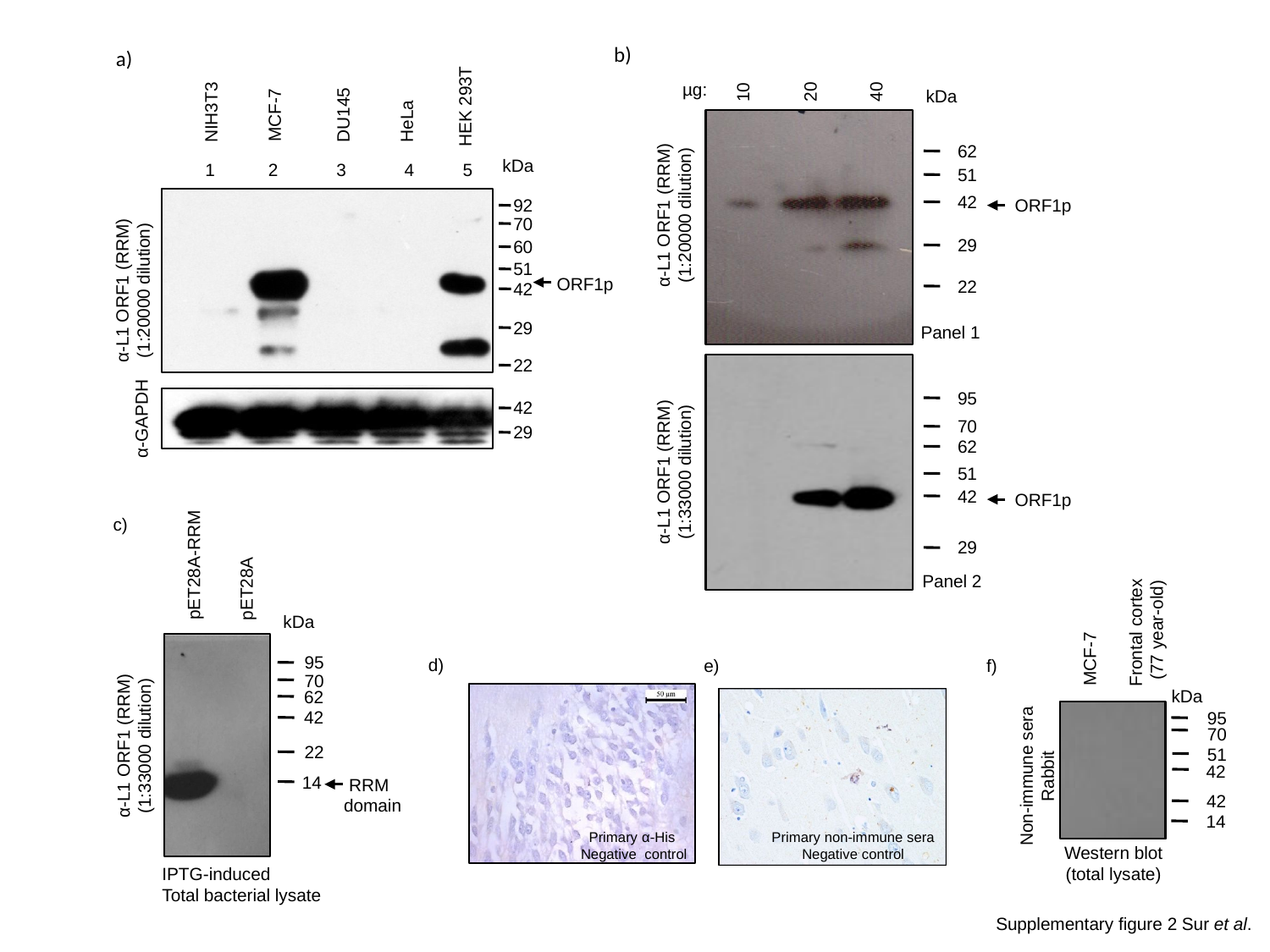

b)
µg:
20
40
10
kDa
62
51
42
α-L1 ORF1 (RRM)
(1:20000 dilution)
 ORF1p
29
22
Panel 1
95
70
62
α-L1 ORF1 (RRM)
(1:33000 dilution)
51
42
 ORF1p
29
Panel 2
a)
HEK 293T
 NIH3T3
 DU145
 MCF-7
 HeLa
kDa
 1 2 3 4 5
92
70
60
51
42
29
22
42
α-GAPDH
29
α-L1 ORF1 (RRM)
(1:20000 dilution)
 ORF1p
c)
pET28A-RRM
pET28A
kDa
95
70
62
42
α-L1 ORF1 (RRM)
(1:33000 dilution)
22
14
 RRM
 domain
IPTG-induced
Total bacterial lysate
Frontal cortex
(77 year-old)
MCF-7
f)
kDa
95
70
51
Non-immune sera
Rabbit
42
42
14
Western blot
(total lysate)
d)
e)
Primary α-His
Negative control
Primary non-immune sera
Negative control
Supplementary figure 2 Sur et al.

## Slide 3
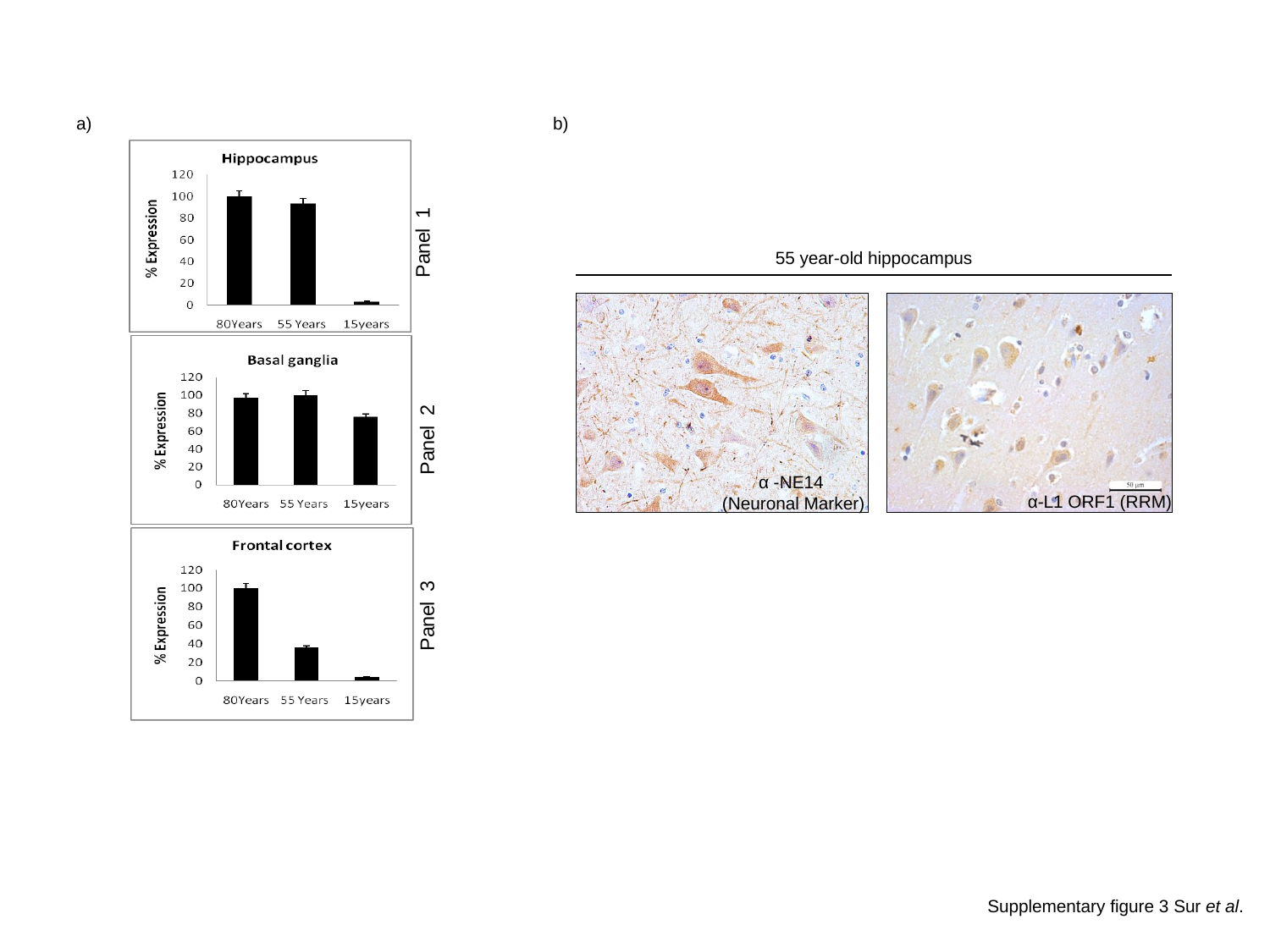

a)
b)
55 year-old hippocampus
α -NE14
 (Neuronal Marker)
α-L1 ORF1 (RRM)
Supplementary figure 3 Sur et al.
