# Supplementary material for: Detection of the LINE-1 retrotransposon RNA-binding protein ORF1p in different anatomical regions of the human brain
Source: Mob DNA. 2017 Nov 22;8:17. doi: 10.1186/s13100-017-0101-4 (PMC5700708; doi:10.1186/s13100-017-0101-4)
Supplement: Supplementary file 1 — Supplementary text and Figures S1-S3. (ZIP 1116 kb) [file 13100_2017_101_MOESM1_ESM.zip › Supple text_ Sur et al RRsub.doc]

**Human L1 ORF1 sequence (RRM domain amino acids 157 to 252 marked dark red)**

```
   atggggaaaaaacagaacagaaaaactggaaactctaaaacgcagagcgcctctcctcct
1   M  G  K  K  Q  N  R  K  T  G  N  S  K  T  Q  S  A  S  P  P
   ccaaaggaacgcagttcctcaccagcaacagaacaaagctggatggagaatgattttgat
21  P  K  E  R  S  S  S  P  A  T  E  Q  S  W  M  E  N  D  F  D
   gagctgagagaagaaggcttcagacgatcaaattactctgagctacgggaggacattcaa
41  E  L  R  E  E  G  F  R  R  S  N  Y  S  E  L  R  E  D  I  Q
   accaaaggcaaagaagttgaaaactttgaaaaaaatttagaagaatgtataactagaata
61  T  K  G  K  E  V  E  N  F  E  K  N  L  E  E  C  I  T  R  I
   accaatacagagaagtgcttaaaggagctgatggagctgaaaaccaaggctcgagaacta
81  T  N  T  E  K  C  L  K  E  L  M  E  L  K  T  K  A  R  E  L
   cgtgaagaatgcagaagcctcaggagccgatgcgatcaactggaagaaagggtatcagca
101 R  E  E  C  R  S  L  R  S  R  C  D  Q  L  E  E  R  V  S  A
   atggaagatgaaatgaatgaaatgaagcgagaagggaagtttagagaaaaaagaataaaa
121 M  E  D  E  M  N  E  M  K  R  E  G  K  F  R  E  K  R  I  K
   agaaatgagcaaagcctccaagaaatatgggactatgtgaaaagaccaaatctacgtctg
141 R  N  E  Q  S  L  Q  E  I  W  D  Y  V  K  R  P  N  L  R  L
   attggtgtacctgaaagtgatgtggagaatggaaccaagttggaaaacactctgcaggat
161 I  G  V  P  E  S  D  V  E  N  G  T  K  L  E  N  T  L  Q  D
   attatccaggagaacttccccaatctagcaaggcaggccaacgttcagattcaggaaata
181 I  I  Q  E  N  F  P  N  L  A  R  Q  A  N  V  Q  I  Q  E  I
   cagagaacgccacaaagatactcctcgagaagagcaactccaagacacataattgtcaga
201 Q  R  T  P  Q  R  Y  S  S  R  R  A  T  P  R  H  I  I  V  R
   ttcaccaaagttgaaatgaaggaaaaaatgttaagggcagccagagagaaaggtcgggtt
221 F  T  K  V  E  M  K  E  K  M  L  R  A  A  R  E  K  G  R  V
   accctcaaaggaaagcccatcagactaacagcggatctctcggcagaaaccctacaagcc
241 T  L  K  G  K  P  I  R  L  T  A  D  L  S  A  E  T  L  Q  A
   agaagagagtgggggccaatattcaacattcttaaagaaaagaattttcaacccagaatt
261 R  R  E  W  G  P  I  F  N  I  L  K  E  K  N  F  Q  P  R  I
   tcatatccagccaaactaagcttcataagtgaaggagaaataaaatactttatagacaag
281 S  Y  P  A  K  L  S  F  I  S  E  G  E  I  K  Y  F  I  D  K
   caaatgttgagagattttgtcaccaccaggcctgccctaaaagagctcctgaaggaagcg
301 Q  M  L  R  D  F  V  T  T  R  P  A  L  K  E  L  L  K  E  A
   ctaaacatggaaaggaacaaccggtaccagccgctgcaaaatcatgccaaaatgtaa
321 L  N  M  E  R  N  N  R  Y  Q  P  L  Q  N  H  A  K  M  -

pcDNAORF1-RRMF
agctcggatccactagtccagtgtggtggaattcaccatggatctacgtctgattggtgtacctgaaagtgatgtggagaatggaaccaagttggaaaacactctgcaggatattatccaggagaacttccccaatctagcaaggcaggccaacgttcagattcaggaaatacagagaacgccacaaagatactcctcgagaagagcaactccaagacacataattgtcagattcaccaaagttgaaatgaaggaaaaaatgttaagggcagccagagagaaaggtcgggttaccctcaaaggaaagcccatcagactaacagcggattaagcggccgctcgagtctagagggcccgcggttcgaacaaaaactcatctcagaagaggatctgaatatgcataccggtcatcatcaccatcaccattgagtttaaacccgctgatcagcctcgactgtgccttctaaaa------
Blue- pcDNA6 myc-His (B) sequence
Green restriction enzyme (EcoRI-GAATTC, NotI GCGGCCGC)
Dark red- ORF1 RRM domain Amino acid position in human ORF1 157-251
taa- stop codon

Codon position                     157  158  159         249  250  251
Human ORF1p RRM domain DNA Seq     CTA  CGT  CTG --------ACA  GCG  GAT
Human ORF1 RRM domain Protein seq   L    R    L  -------- T    A    D

pET28a-ORF1-RRM (Cloned between EcoRI and NotI sites)

   atgggcagcagccatcatcatcatcatcacagcagcggcctggtgccgcgcggcagccat
1   M  G  S  S  H  H  H  H  H  H  S  S  G  L  V  P  R  G  S  H
   atggctagcatgactggtggacagcaaatgggtcgcggatccgaattcaccatggatcta
21  M  A  S  M  T  G  G  Q  Q  M  G  R  G  S  E  F  T  M  D  L
   cgtctgattggtgtacctgaaagtgatgtggagaatggaaccaagttggaaaacactctg
41  R  L  I  G  V  P  E  S  D  V  E  N  G  T  K  L  E  N  T  L
   caggatattatccaggagaacttccccaatctagcaaggcaggccaacgttcagattcag
61  Q  D  I  I  Q  E  N  F  P  N  L  A  R  Q  A  N  V  Q  I  Q
   gaaatacagagaacgccacaaagatactcctcgagaagagcaactccaagacacataatt
81  E  I  Q  R  T  P  Q  R  Y  S  S  R  R  A  T  P  R  H  I  I
   gtcagattcaccaaagttgaaatgaaggaaaaaatgttaagggcagccagagagaaaggt
101 V  R  F  T  K  V  E  M  K  E  K  M  L  R  A  A  R  E  K  G
   cgggttaccctcaaaggaaagcccatcagactaacagcggattaa
121 R  V  T  L  K  G  K  P  I  R  L  T  A  D STOP


Light blue- vector sequence
Dark red-ORF1 RRM domain
taa- stop codon
```

**Compute pI/Mw ( ExPASY Bioinformatics Resource portal)**

**Theoretical pI/Mw (average) for the user-entered sequence:**

```
        10         20         30         40         50         60
MGSSHHHHHH SSGLVPRGSH MASMTGGQQM GRGSEFTMDL RLIGVPESDV ENGTKLENTL

        70         80         90        100        110        120
QDIIQENFPN LARQANVQIQ EIQRTPQRYS SRRATPRHII VRFTKVEMKE KMLRAAREKG

       130
RVTLKGKPIR LTAD
```

Theoretical pI/Mw: 10.62 / 15174.32

Bacterial expressed hORF1 RRM domain protein is 134 amino acid (AA 1-38 from vector and 39-134 from RRM) in length with MW 15kDa.

**Details of individuals from where samples were collected:**

| Sample | Age | Sex | PMI (hours) | Available section |
| --- | --- | --- | --- | --- |
| 1 | 80 | Female | 16.45 | FT, HP, BG, TH,MD, MG,SP |
| 2 | 77 | Male | 13.45 | FT |
| 3 | 55 | Female | 5.3 | FT,HP,BG |
| 4 | 15 | Male | 20.3 | FT, HP, BG, TH,MD, MG |

PMI-Post-Mortem Interval, FT= Frontal Cortex, HP= Hippocampus,

BG=Basal Ganglia, TH= Thalamus, MG= Medulla Oblongata

SP= Spinal Cord

**B) Immunization protocol:**

The Rabbit was kept and maintained in Jawaharlal Nehru University (JNU) animal facility (JNU, New Delhi India) as per institute animal use guidelines. The recombinant antigen (human ORF1 RRM domain peptide) was mixed with equal volume of Complete Freund's Adjuvent (CFA) and passed through a glass syringes to make an emulsion. The emulsion was injected subcutaneously at four locations according to the following schedule. For subsequent immunization (Booster), Incomplete Freund's Adjuvant was used. Blood was collected and kept at 37°C for one hour and then transferred to 4°C overnight. The coagulated blood was centrifuged at 8000xg/4C/20 minutes and the supernatant containing the serum was collected and stored in aliquots at -80°C.

**Parameter:**

| **Parameter** | **Rabbit** |
| --- | --- |
| Strain | NZW |
| Number of animal used | 1 |
| Amount of antigen Primary Booster | Primary : 150-200 µg Secondary: 100-150 µg |
| Volume of antigen with adjuvant | 1.5 ml |
| Amount of Bleed | 3-4 ml (test bleed)  10-12 ml (final bleed) |

**Immunization schedule:**

| **Day** | **Activity** |
| --- | --- |
| 3 days before immunization | Pre bleed ( checked by western for non- specific signal) |
| 0 | Primary immunization |
| 14 | Test bleed (1) |
| 21 | 1st booster |
| 35 | Test bleed (2) |
| 42 | 2nd booster |
| 56 | Final bleed |

C). Figure legend:

**Supplementary Figure 1:** Scheme of human L1ORF1p. The L1-ORF1p RRM domain cloned earlier in pcDNA6 (20, 51) was swapped in bacterial expression vector pET28a. The bacterial expressed RRM domain is 134 amino acids in length with MW ~15 kDa.

**Supplementary Figure 2:** Characterization of α-human L1 ORF1p (RRM) using western blot analysis. **(a)** Detection of endogenous ORF1p (~40kDa) and an extra lower molecular weight band (~25kDa) in total lysates from MCF-7 and HEK 293T cell lines using α-human L1 ORF1p (RRM) at 1:20,000 dilution. Lane 1: NIH3T3 (mouse embryonic fibroblast), Lane 2: MCF-7 (human breast cancer), Lane 3: DU145 (human prostate cancer cell line), Lane 4: HeLa (human epithelial cancer) and Lane 5: HEK293T (human embryonic kidney). **Panel 2**: immunoblot with α-GAPDH. **(b)** Different amounts of total lysate (10 µg, 20 µg and 40 µg) from HEK 293T cells were separated on SDS-PAGE followed by immunoblotting with α-human L1 ORF1p (RRM) using two different dilutions - 1:20,000 dilution (top panel-1) and 1:33,000 (bottom panel-2), respectively. **(c)** Western blot analysis of IPTG induced bacterial total lysate using α-human L1 ORF1p (RRM). **(d)** IHC negative control : primary α-His (raised in mouse) and secondary α-mouse . **(e)** IHC negative control : primary non-immune sera (rabbit) and secondary α-rabbit was used to stain hippocampus of 77 year old. **(f)** Western blot analysis of total lysate obtained from MCF-7 (lane 1) and frontal cortex 77 year old (lane 2) using rabbit non-immune sera as primary antibody.

**Supplementary figure 3** **(a)** Quantification of DAB signal intensities from different regions of brain. Values were calculated and plotted using ImmunoRatio software [53].The sample which showed highest expression among three samples (80 year, 55 year and 15 year) was considered 100% expression. **(b)** IHC staining analysis of 55 year old hippocampus section using α- Neurofilament (NE14) as neuronal marker . Same section stained with α-human L1 ORF1p (RRM) was used as control.
